# Bone Regeneration Using Dentin Matrix Depends on the Degree of Demineralization and Particle Size

**DOI:** 10.1371/journal.pone.0147235

**Published:** 2016-01-21

**Authors:** Takamitsu Koga, Tokutaro Minamizato, Yosuke Kawai, Kei-ichiro Miura, Takashi I, Yuya Nakatani, Yoshinori Sumita, Izumi Asahina

**Affiliations:** Department of Regenerative Oral Surgery, Unit of Translational Medicine, Nagasaki University Graduate School of Biomedical Sciences, Nagasaki, Japan; Second University of Naples, ITALY

## Abstract

**Objectives:**

This study aimed to examine the influence of particle size and extent of demineralization of dentin matrix on bone regeneration.

**Materials and Methods:**

Extracted human teeth were pulverized and divided into 3 groups according to particle size; 200, 500, and 1000 μm. Each group was divided into 3 groups depending on the extent of demineralization; undemineralized dentin (UDD), partially demineralized dentin matrix (PDDM), and completely demineralized dentin matrix (CDDM). The dentin sample was implanted into rat calvarial bone defects. After 4 and 8 weeks, the bone regeneration was evaluated with micro-CT images, histomorphometric and immunohistochemical analyses. Osteoblasts were cultured on UDD and DDM to evaluate the cell attachment using electron microscope.

**Results:**

Micro-CT images and histological observation revealed that CDDM had largely resorbed but UDD had not, and both of them induced little bone formation, whereas all particle sizes of PDDM induced more new bone, especially the 1000 μm. Electron microscopic observation showed osteoblasts attached to DDM but not to UDD.

**Conclusions:**

PDDM with larger particle size induced prominent bone regeneration, probably because PDDM possessed a suitable surface for cell attachment. There might be an exquisite balance between its resorption and bone formation on it. PDDM could be considered as a potential bone substitute.

## Introduction

The recent popularity of implant dentistry has led to an increasing demand for alveolar bone regeneration. Autogenous bone grafting is still the gold standard for bone augmentation because of its excellent osteoinductivity and osteoconductivity [1, 2], but it has some impediments such as limited availability, donor site morbidity, and also high resorption rates of up to 50% [3]. Alternative graft materials including allografts [4–6], xenografts [7, 8], and alloplastic bone grafts [9, 10] are clinically used, but they have disadvantages such as high cost and limited osteoinductivity. Among these, demineralized freeze-dried bone allografts (DFDBAs) have been widely used for alveolar bone augmentation [5, 6] because of their natural structure and inclusion of growth factors such as bone morphogenetic proteins (BMPs) [11], ever since successful bone augmentation with DFDBAs in humans was first shown in 1981 [12]. However, DFDBA carries the risk of disease transmission. Thus, development of an alternative material that overcomes these shortcomings is expected.

The structure and composition of dentin are similar to that of bone, consisting of collagen (20%), hydroxyapatite (70%), and body fluid (10%) in weight [13]; so it is thought to have significant osteoconductivity. Furthermore, dentin matrix has some osteoinductivity because it contains BMPs [13]. Thus, dentin or dentin matrix is expected to serve as a bone substitute. Some studies have shown that mineralized dentin matrix possesses excellent biocompatibility, but is less effective in bone formation than bone-derived products [14–16]. However, several animal studies showed that demineralized dentin matrix (DDM) is not only biocompatible but also osteoinductive, similar to demineralized bone matrix [17–22]. Gomes et al. first reported that the clinical application of autogenous sliced DDM to the extraction socket of mandibular third molar showed slightly better healing of the sockets [23]. Kim et al. applied both mineralized dentin and demineralized dentin matrix particles in dental implant surgery and reported successful bone regeneration [24, 25].

We sometimes encounter cases that require the extraction of teeth prior to oral rehabilitation using dental implants. It is beneficial if we can utilize these extracted teeth, which are usually discarded, as autogenous grafting material as the operative procedure to harvest such tissue could be avoided. However, there is limited information about the suitable form of dentin matrix which can be used as a bone substitute. Therefore, we aimed to clarify the appropriate degree of demineralization and particle size of dentin matrix for bone regeneration in this animal study.

## Materials and Methods

### Preparation of Dentin Particles

The Ethics Committee for Clinical Study of the Nagasaki University Hospital approved this research (No. 11052368). Extracted teeth from healthy humans were collected from the Oral Surgery Clinic at Nagasaki University Hospital with the informed consent of patients. Either vital or non-vital extracted teeth, from which soft tissues, calculus, crown restorations and root fillings had been removed, were crushed with a percussion mill (Polymix^®^ PX-MFC 90 D, Kinematica AG, Switzerland). The particles were collected and passed through a series of sieves (180 μm-212 μm, 425 μm-600 μm, 800 μm-1200 μm mesh) and separated into a 200 μm group (ranging from 180 to 212 μm in diameter), 500 μm group (425 to 600 μm), and 1000 μm group (800 to 1200 μm), and were washed thoroughly in 1.0 M sodium chloride.

### Preparation of Demineralized Dentin Matrix

Dentin particles were demineralized in 2% HNO_3_ and separated into three groups according to the degree of demineralization; undemineralized dentin (UDD), partially demineralized dentin matrix (PDDM) which was 70% decalcified, and completely demineralized dentin matrix (CDDM). The decalcification time for each size of dentin particles was determined by measuring the concentration of eluted Ca in solution and residual Ca in the dentin over time using Calcium E test kit (Wako, Japan). Then, each DDM particle was extensively rinsed twice in 0.1 M Tris-HCl (pH 7.4), for 10 minutes.

### Implantation and harvest of DDM in rat calvarial bone defect

This study was carried out in strict accordance with the recommendations in the Guide for the Care and Use of Laboratory Animals of the National Institute of Health. The Animal Research Committee of Nagasaki University approved the study protocol (No. 1008020869). All sections of this report adhere to the ARRIVE Guidelines for reporting animal research. A completed ARRIVE guidelines checklist is included in the S1 Checklist. In this study, 100 8-week-old male Fischer rats (F344, Clea Japan Inc., Japan) (240±20 g) were used. The rats were housed with an inverse 12 hours day-night cycle with lights on at 7:00 and off at 19:00 in a temperature (21±2°C) and humidity (55±10%) controlled room. They had access to rat maintenance food and water. All surgical procedures were performed under sodium pentobarbital anesthesia, and all efforts were made to minimize suffering. The rat calvarial bone defect model was used in this study because it is a standard inexpensive assay for evaluating new bone formation in an osseous defect [26]. The experimental rats were anesthetized with intraperitoneal sodium pentobarbital (25 mg/kg). A skin incision was made at the middle of the parietal region, and the periosteum was ablated. A 6 mm calvarial bone defect was made with a trephine operating at 1500 rpm or less under continuous saline buffer irrigation, and UDD, PDDM or CDDM were implanted into the defect in a random manner. Each of the 9 experimental groups used a graft material which was made from 20 mg of dentin particles. A control defect was left empty. In this study, five rats were used for each experimental group. After the implantation, the ablated periosteum was repositioned, and the skin was sutured. After 4 and 8 weeks, the rats were euthanized by cervical dislocation under anesthesia with ether, and the specimens including the implant were harvested.

### Radiographic analysis

The specimens were scanned using a micro-computed tomography (μCT) system (Softex CMB-2, SOFTEX Co, Japan) under standardized conditions. Using three-dimensional (3D) structural analysis software (TRI/3D-BON, Ratoc System Engineering, Japan), 3D μCT images of calvarium were reconstructed and morphometrically analyzed.

### Histological and immunohistochemical analysis

After the radiographic analysis, the specimens were prepared using standard procedures for histological and immunohistochemical analysis. Briefly, the specimens were fixed with 4% paraformaldehyde in 0.1M PBS (pH 7.4), and decalcified in 0.5M EDTA (pH7.8) for 10 days. After decalcification, each specimen was transversally divided into two blocks, exactly along the center of the original surgical defect, processed and embedded in paraffin, and transverse serial sections of 5 μm thickness were prepared. The sections were stained with hematoxylin and eosin (HE) and tartrate resistant acid phosphate (TRAP). Osteocalcin was detected immunohistochemically. For immunohistochemical analysis, staining without a primary antibody was performed as negative control. Briefly, the sections were blocked with 0.1% BSA in PBS and incubated for 20 min. A primary antibody (sc-365797, Santa Cruz Biotechnology, USA) at a dilution of 1:100 was added to each section and incubated at 37°C for 30 min. The ABC complex (VECTASTAIN^®^ Elite ABC kit, Vector Laboratories, USA) was applied to the sections after incubation with a biotinylated secondary antibody (VECTASTAIN^®^ Elite ABC kit, Vector Laboratories, USA). 3, 3’-diaminobenzidine (ImmPACT DAB, Vector Laboratories, USA) was used as a chromagen, and the sections were counterstained with light Methyl Green. These sections were examined by photomicroscope (Nikon digital sight DS-U2, Nikon Co, Japan).

### Quantitative micrograph analysis

Light micrographs of the sections stained with HE were used for histomorphometric measurements. Four sections from each specimen were made, and photographs projecting the overall defect were taken. The percentage of newly formed bone in the defect was calculated as the area of newly formed bone per area of the defect originally created by trephination (n-Bone%). Likewise, the percentage of remaining implants in the defect was calculated as the area of remaining implants per area of the defect originally created by trephination (r-Imp%). These areas were quantified on a computer using NIH Imaging (Image-J).

### Statistical analysis

One-way analysis of variance (ANOVA) with the Tukey-Kramer test was used to compare the means between groups. Differences were considered significant at p<0.05 or p<0.01.

### Osteoblasts culture on the UDD and DDM plates

Primary osteoblasts were isolated from newborn calvaria by sequential digestion with 0.1% collagenase and 0.2% dispase. The osteoblastic cells from the third to fifth fraction were pooled, and expanded on UDD and DDM plate (10 × 5 × 2 mm) in αMEM, 10%FBS, and 1% penicillin / streptomycin using 48-well plates. The cells were grown and observed for 10 days with medium changes every 3 days, and the cell adhesion to the matrix was then assessed with scanning electron microscopy (TM-1000 Miniscope^®^, Hitachi, Japan) [27].

## Results

### Time course of demineralization of dentin particles

**Fig 1:**
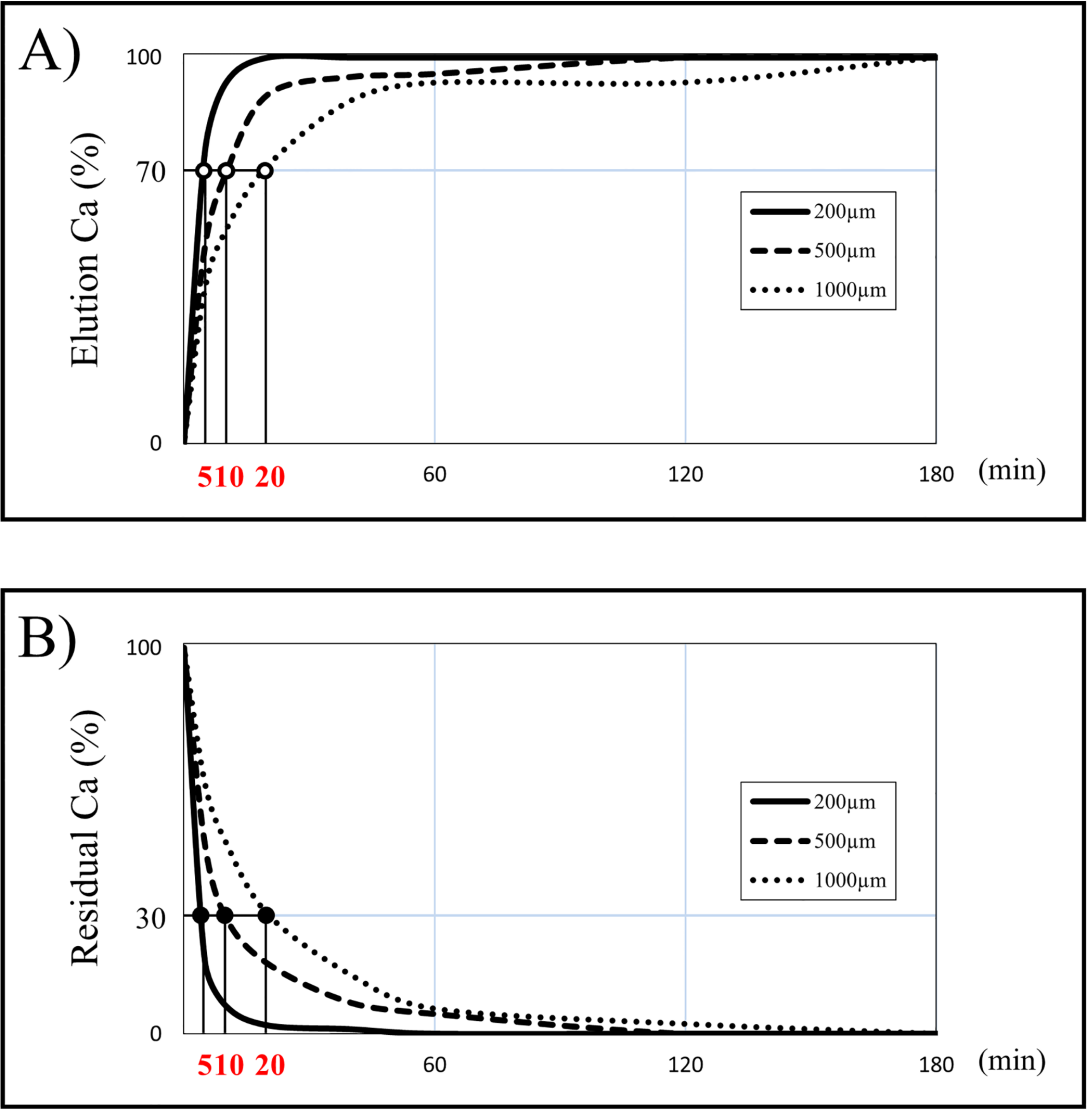
The percentage of elution Ca in solution and residual Ca in dentin in the process of demineralization by 2% HNO_3_. The time setting for the duration of demineralization was based on the results of the percentage of elution Ca in solution and residual Ca in the dentin which were measured over time using the Calcium E test kit in order to estimate the degree of demineralization.

When preparing DDM, the concentration of eluted Ca in solution (Fig 1A) and residual Ca in dentin (Fig 1B) were monitored for each particle size of dentin during the demineralization process with 2% HNO_3_. In 200 μm particles, the eluted Ca and also residual Ca showed a steep inclination curve in 20 minutes, and reached a plateau in 60 minutes. In 500 μm and 1000 μm particles, it showed a gentle curve compared with 200 μm particles, and reached a plateau in 120 and 180 minutes, respectively. According to these results, 70% demineralization of dentin took 5, 10, and 20 minutes and complete demineralization of dentin took 60, 120, and 180 minutes for 200, 500 and 1000 μm particles, respectively (Table 1).

**Table 1:** The time for preparation of dentin matrix.

Particle sizes	Degree of demineralization (min)		
	UD	PDDM	CDDM
200μm	0	5	60
500μm	0	10	120
1000μm	0	20	180

### μCT analysis

**Fig 2:**
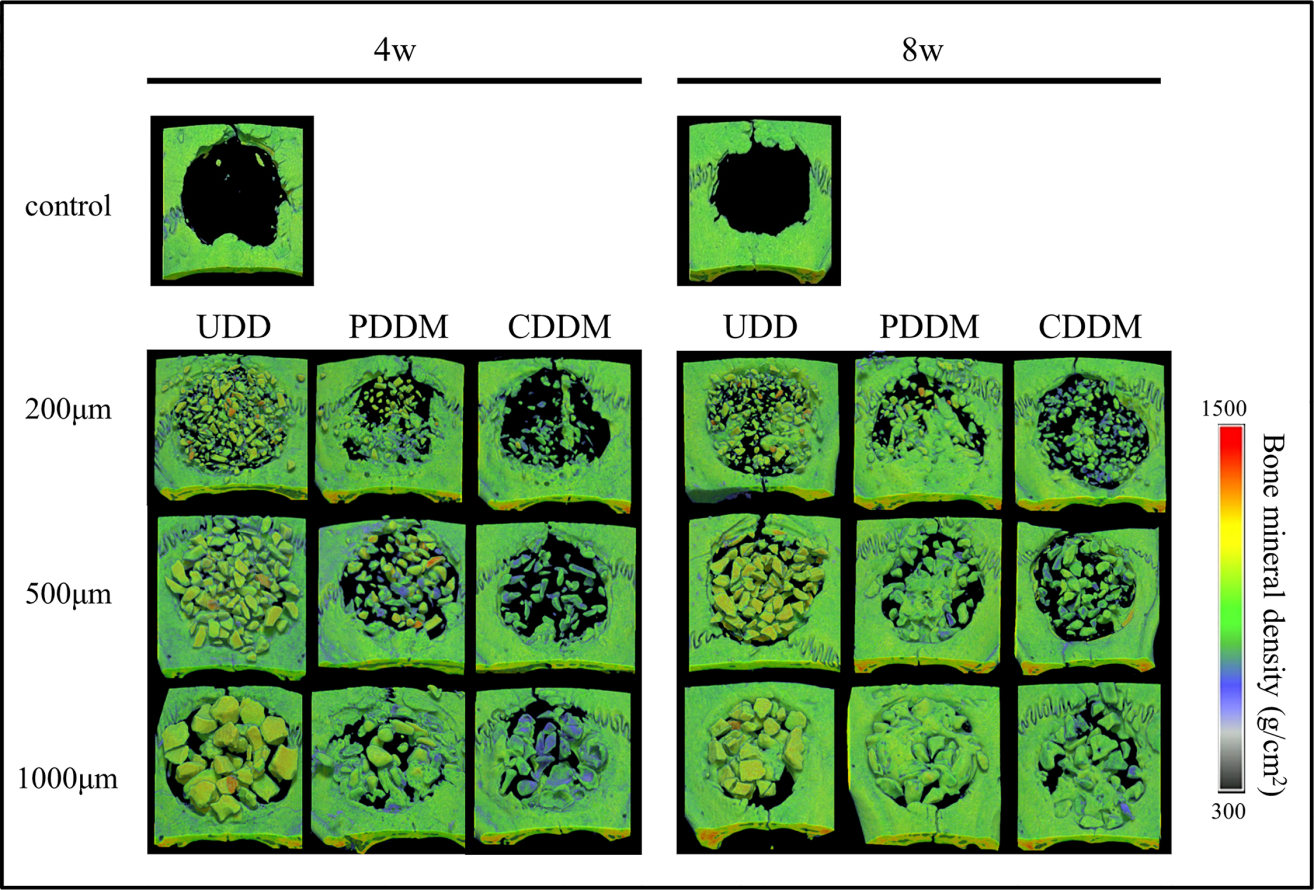
Radiographic analysis of specimens using μCT. New bone formation around PDDM is enhanced in all particle sizes compared with CDDM in a time-dependent manner. The results of 1000 μm PDDM are especially noteworthy. On the contrary, almost all samples of UDD, with the exception of 1000 μm at 8 weeks, resulted in the defect being mostly occupied with dentin particles.

Images of μCT are shown in Fig 2. In terms of the degree of demineralization, empty spaces among the particles were frequently observed in all sizes of CDDM implant at both 4 and 8 weeks after surgery. However, the space tended to be filled with newly formed bone in all sizes of PDDM implant, especially at 8 weeks after surgery. In terms of the particle size, the larger-sized particles tended to induce more new bone formation. On the contrary, the particles of UDD remained in the defects in all the samples and the spaces among UDD particles remained except in 1000 μm at 8 weeks.

### Histological findings of HE-stained sections at low-power magnification view

The arrowheads indicate the edge of the original bone defect.

**Fig 3:**
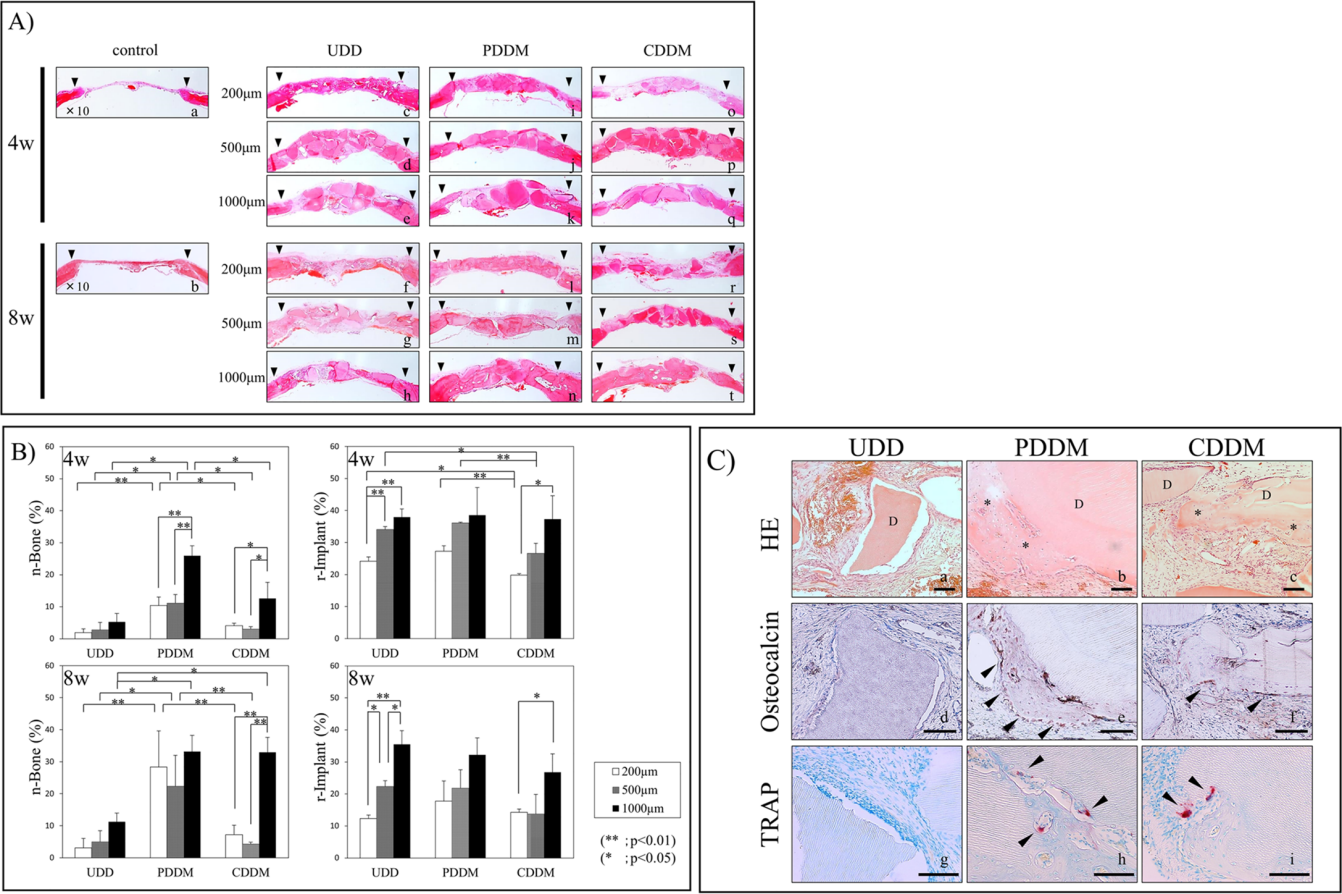
A) Histological findings of HE stained sections at low-power magnification. The black arrowhead represents the edge of the cortical bone defect. In the UDD group, the area of newly formed bone was limited around the UDD in almost all sections (A-c, d, e, f, g). In the PDDM group, new bone formation surrounding the PDDM was evident at 4 weeks (A-i, j, k), especially in the 1000 μm samples (A-k). At 8 weeks, the newly formed bone is present in variable extents around PDDM, and complete closure in the surgical defect by consecutive new bone formation with PDDM is confirmed from end to end of the defect (A-l, m, n). In the CDDM group, the new bone formation is confirmed to be not only surrounding the CDDM, but also in the gaps between CDDMs (A-t) at 8 weeks in the 1000 μm samples. B) Quantitative micrograph analysis. Histomorphometric findings regarding n-Bone% and r-Imp% are shown. At 4 weeks, significant difference of n-Bone% is observed between the PDDM and other groups in each particle size. Within the PDDM and CDDM groups, the n-Bone% of 1000 μm particles showed a significant difference compared with those of the 200 μm and 500 μm groups. A significant difference in r-Imp% was observed between the CDDM and the other groups in the 200 μm and 500 μm samples. At 8 weeks, a significant difference in the n-Bone% was observed between the PDDM and the other groups in the 200 and 500 μm samples. In the 1000 μm group, the n-Bone% of PDDM and CDDM shows a significant increase in comparison with that of UDD. C) Histological findings of 1000 μm UDD, PDDM and CDDM at 4 weeks. This is the high-power magnification view of 1000 μm UDD, PDDM and CDDM sections stained with HE (C-a, b, c), Osteocalcin (C-d, e, f) and TRAP (C-g, h, i) at 4 weeks. New bone formation (* in C-b, c) surrounding PDDM and CDDM (D in C-b, c) was evident, and they were amalgamated. The maturation of new bone with many lacunae containing osteocytes (C-b) and some lining cells on the surface of the newly formed bone was evident. The presence of Osteocalcin–positive cells (arrowheads in C-e, f) and some TRAP-positive multinucleated giant cells (arrowheads in C-h, i) with Howship’s lacunae in that area was confirmed. Bars = 100 μm.

In the control, little bone formation was observed, and only at the edges of the defects both at 4 and 8 weeks (Fig 3A-a, b).

The area of newly formed bone was limited to only around UDD in almost all samples (Fig 3A-c, d, e, f, g). Newly formed bone was prominent in some areas only in a 1000 μm sample (Fig 3A-h). There were very few osteoblasts and osteoclasts in these specimens, and the configuration of UDD did not show any obvious change.

New bone formation surrounding PDDM along with well organized connective tissue and osteoid like tissue was evident after 4 weeks (Fig 3A-i, j, k), especially in the 1000 μm sample (Fig 3A-k). The newly formed bone and PDDM were amalgamated and complete closure of the defects with new bone was observed after 8 weeks (Fig 3A-l, m, n).

As for CDDM, a limited amount of new bone formation was observed around the 1000 μm sample at 4 weeks (Fig 3A-o, p, q). After 8 weeks, new bone formation was apparent and almost all CDDM was replaced by newly formed bone in the 1000 μm CDDM samples (Fig 3A-t). On the contrary, little bone formation was observable in the 200 μm and 500 μm samples; some matrices had resorbed and the defects were filled with connective tissue in the 200 μm samples (Fig 3A-r, s).

### Histomorphometric analysis

The amount of newly formed bone (n-Bone%) and remaining implants (r-Imp%) were quantitatively measured by histomorphometric analysis (Fig 3B). At 4 weeks, the mean n-Bone% of the 200 μm, 500 μm, and 1000 μm samples of UDD were 1.9%, 2.7%, and 5.6%; those of the 200 μm, 500 μm, and 1000 μm of PDDM were 10.4%, 11.1%, and 26.0%; and those of the 200 μm, 500 μm, and 1000 μm of CDDM were 4.1%, 2.1%, and 12.6%, respectively. A significant difference in n-Bone% was observed between PDDM and the other groups for each particle size. Within the PDDM and CDDM groups, the n-Bone% of the 1000 μm samples showed a significant difference with those of the 200 μm and 500 μm samples. The mean r-Imp% of the 200 μm, 500 μm, and 1000 μm samples of UDD were 24.1%, 34.1%, and 37.9%, respectively; those of the 200 μm, 500 μm, and 1000 μm of PDDM were 27.3%, 36.1%, and 38.5%, respectively; and those of the 200 μm, 500 μm, and 1000 μm of CDDM were 19.8%, 26.6%, and 37.3%, respectively. A significant difference in r-Imp% was observed between the CDDM and the other groups in the 200 μm and 500 μm samples.

After 8 weeks, the mean n-Bone% of the 200 μm, 500 μm, and 1000 μm samples of UDD were 3.1%, 5.0%, and 11.2%, respectively; those of the 200 μm, 500 μm, and 1000 μm of PDDM were 28.3%, 22.3%, and 33.2%, respectively; and those of the 200 μm, 500 μm, and 1000 μm of CDDM were 7.2%, 4.3%, and 33.0%, respectively. A significant difference in n-Bone% was observed between PDDM and the other groups in the 200 μm and 500 μm samples. In the 1000 μm sample, the n-Bone% of PDDM and CDDM showed a significant increase compared with that of UDD. The mean r-Imp% of the 200 μm, 500 μm, and 1000 μm samples of UDD were 12.4%, 22.3%, and 35.5%, respectively; those of the 200 μm, 500 μm, and 1000 μm of PDDM were 17.8%, 21.8%, and 32.3%, respectively; and those of the 200 μm, 500 μm, and 1000 μm of CDDM were 14.3%, 13.7%, and 26.8%, respectively. There was no significant difference in r-Imp% among UDD, PDDM and CDDM groups in each particle size.

### Histological findings of 1000 μm UDD, PDDM and CDDM at 4 weeks

Since the new bone formation of 1000 μm PDDM was superior to those of the other sizes, we further investigated the early stage of bone regeneration in 1000 μm UDD, PDDM and CDDM histologically. Fig 3C shows HE (Fig 3C-a, b, c), osteocalcin (Fig 3C-d, e, f) and TRAP (Fig 3C-g, h, i) stained sections with high-power magnification. New bone formation (* in Fig 3C-b, c) surrounding PDDM and CDDM (D in Fig 3C-b, c) was evident, and they were amalgamated. The maturation of new bone with many lacunae containing osteocytes (Fig 3C-b, c) and some lining cells on the surface of the newly formed bone could be noted. Newly formed bone was lined with osteocalcin–positive cells (arrowheads in Fig 3C-e, f). Interestingly, TRAP-positive multinucleated giant cells (arrowheads in Fig 3C-h, i) were visible both on PDDM and CDDM, but not on UDD.

### Osteoblast culture on the surface of UDD and DDM

**Fig 4:**
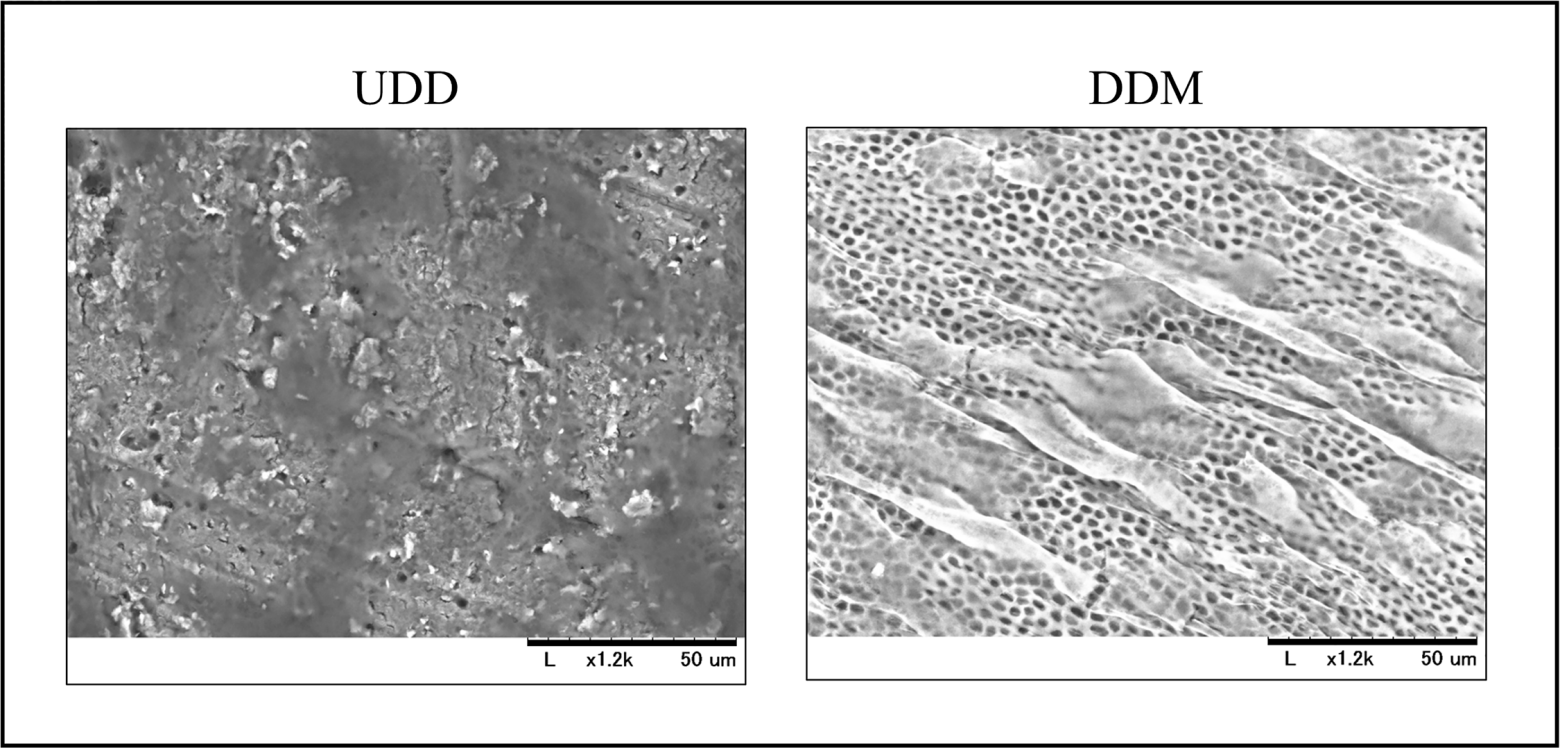
Osteoblast culture on the surface of UDD and DDM. SEM micrographs show that cell morphology is sensitive to surface nanostructure. Dentinal tubes are clearly observed on the DDM surface, but not on the UDD surface. Osteoblasts were cultured on the surfaces of UDD and DDM; however, osteoblast attachment was observed only on the surface of DDM.

Scanning electron microscope (SEM) micrographs are shown in Fig 4. Dentinal tubules were clearly observed on the DDM surface, which was smooth, whereas the UDD surface was uneven and small debris were observed. Osteoblasts were well attached and spread out only on the surface of DDM. There was no difference in cell attachment between PDDM and CDDM.

## Discussion

We demonstrated that DDM, especially PDDM, was an effective bone substitute for the regeneration of rat calvarial bone defects in this study. UDD showed much less bone regenerative activity as shown in Fig 3, although freeze-dried bone allograft (FDBA) has bone conductivity and is widely applied in clinical settings [28]. This may be because the fine structure of dentin is different from that of bone [29] though it has similar composition with bone [13]. Almost no osteoblasts were seen attached to the surface of UDD, while many osteoblasts were attached and spread on the surface of DDM, as demonstrated in electron-microscopic observation (Fig 4). The exposure of dense collagen fibers may be favorable for the cell attachment. Another advantage of demineralization for bone regeneration is the following. It is well known that dentin contains several non-collagenous proteins including osteogenic growth factors such as BMPs [30–32]. Once dentin is demineralized, the dentinal tubule would become wider and serve as a channel for releasing essential proteins [22], which may promote growth and differentiation of osteoblasts. Turonis et al. reported that human DFDBA appears to significantly enhance osseous wound healing in rat calvarium compared to FDBA [33]. They pointed out that the mineralized nature of FDBA may have impeded the remodeling process by limiting the exposure of the underlying growth factors.

It is interesting that PDDM had superior bone regenerative activity than CDDM especially in the early stage of bone regeneration. Histological observation showed that the reduction in size of implanted particles and the occupation of the spaces with connective tissue were prominent in CDDM implantation, especially in smaller particles, compared to PDDM implantation as shown in Figs 2 and 3A. These observations were supported by quantitative analysis of new bone formation and remaining dentin matrix, especially in smaller particles observed at 4 weeks (Fig 3B). This suggests that resorption of CDDM occurred faster than bone formation both by osteoconduction and osteoinduction on CDDM. Collagen is mostly absorbed by enzymatic digestion [34]. It is noteworthy that TRAP-positive osteoclastic cells were seen on the surface of PDDM and CDDM but not on UDD. This implies that collagen fibrils were resorbed not only enzymatically but also phagocytically. This osteoclastic resorption may release the factors containing mineralized matrix, which initiate the coupling with osteoblastic bone formation. It is possible that PDDM contains more growth factors that promote osteogenesis than CDDM does, since many non-collagenous proteins are released from dentin matrix during the process of demineralization. This may account for the prominent bone formation in PDDM than CDDM.

In terms of particle size, 800 to 1200 μm showed superior results in bone regeneration than the smaller sizes, 180 to 212 μm and 425 to 600 μm, in all groups of UDD, PDDM and CDDM in the present study. Several studies have focused on the influence of particle size of graft materials on bone regeneration. However, there is no consensus regarding the optimum particle size of graft materials for bone regeneration, since graft material and also site characteristics varied in each study. Many studies using inorganic material such as bovine bone particles and FDBA resulted in superior bone formation, and also more absorbability with smaller particles around 300 μm in size [35–37]. On the other hand, studies using fresh autogenous bone particles or DFDBA showed superior bone formation with larger particles around 1000 μm in size [38, 39]. In these studies, small particles were resorbed before the initiation of bone formation, and a similar phenomenon was observed in the CDDM group in our study. Mesenchymal cells including osteoblasts and osteoprogenitor cells might attach to the surface of larger CDDM particles before CDDM is completely resorbed. The attachment of these cells may prevent resorption and initiate bone formation. The new bone formation in 1000 μm CDDM gradually caught up with that of PDDM after 8 weeks (Fig 3B). Consequently, the optimal absorption rate of substrate is a crucial factor for superior bone regeneration. Thus, small size particle is suitable for highly mineralized tissue such as FDBA but not for PDDM and CDDM. Alternatively, the balance between resorption of matrix and bone formation on matrix is critical for optimal bone regeneration, and PDDM is thought to have optimal conditions.

PDDM with large particle size (1000 μm) showed superior bone regeneration in this rat calvarial bone defect model. Although 70% demineralization was used for PDDM, different degrees of demineralization, for example only surface demineralization, may show superior results. In terms of particle size, it is possible that a mixture of particles with variable sizes may have better results. Further studies are necessary to determine the most suitable conditions of demineralization and particle size for clinical application in implant dentistry.

It is beneficial to utilize the extracted teeth as a bone substitute in implant dentistry, even though there are limited cases with available teeth, in addition to a limited volume. However, there is no risk of transmitting diseases as it is autogenous tissue and no additional surgery is needed to harvest tissue since unwanted teeth are utilized. In the present study, we suggest that partial demineralization was ideal for bone regeneration. Partial demineralization clinically has an advantage, such as decontamination of attached bacteria, and also it takes little time, which enables immediate application of an extracted tooth. We recommend the application of PDDM as a bone substitute in cases where teeth indicated for extraction are available.

## Conclusions

We conclude that PDDM with a large particle size of around 1000 μm induced prominent bone regeneration of bony defects since PDDM possessed a suitable surface for cell attachment. There is also an exquisite balance between its resorption and bone formation; as such, PDDM could be considered as an option for bone substitutes. PDDM could be clinically applicable for alveolar bone regeneration in relatively small bone defects, as in socket preservation, immediate implant placement and sinus floor augmentation with crestal approach. It will not be long before it can be clinically applied because it is easy to prepare and the precedent clinical studies have approved its safety as an autogenous graft.

## Supporting Information

S1 ARRIVE Checklist ARRIVE guidelines checklist. (PDF)
